# Water oxidation by Ferritin: A semi-natural electrode

**DOI:** 10.1038/s41598-019-47661-z

**Published:** 2019-08-08

**Authors:** Zahra Abdi, Robabeh Bagheri, Zhenlun Song, Mohammad Mahdi Najafpour

**Affiliations:** 1 Department of Chemistry, Institute for Advanced Studies in Basic Sciences (IASBS), Zanjan, 45137-66731 Iran; 2 Surface Protection Research Group, Surface Department, Ningbo Institute of Materials Technology and Engineering, Chinese Academy of Sciences, 519 Zhuangshi Road, Ningbo, 315201 China; 3 Center of Climate Change and Global Warming, Institute for Advanced Studies in Basic Sciences (IASBS), Zanjan, 45137-66731 Iran; 4 Research Center for Basic Sciences & Modern Technologies (RBST), Institute for Advanced Studies in Basic Sciences (IASBS), Zanjan, 45137-66731 Iran

**Keywords:** Biocatalysis, Electrocatalysis, Heterogeneous catalysis

## Abstract

Ferritin is a protein (ca. 12 nm) with a central pocket of 6 nm diameter, and hydrated iron oxide stored in this central cavity of this protein. The protein shell has a complicated structure with 24 subunits. Transmission electron microscopy images of ferritin showed nanosized iron oxides (ca. 4–6 nm) in the protein structure. In high-resolution transmission electron microscopy images of the iron core, d-spacings of 2.5–2.6 Å were observed, which is corresponded to d-spacings of ferrihydrite crystal structure. Our experiments showed that at pH 11, the modified electrode by this biomolecule is active for water oxidation (turnover frequency: 0.001 s^−1^ at 1.7 V). Using affected by bacteria, we showed that Fe ions in the structure of ferritin are critical for water oxidation.

## Introduction

Hydrogen production by water splitting is an interesting and promising strategy to store of sustainable but intermittent energies^1–3^. Although the cathodic reaction is of major importance in water splitting, water oxidation in the anode is a limitation for water splitting in according to large overpotential and stability issues^4–9^. Ruthenium and iridium-based catalysts show efficient activity for water oxidation^10,11^, but these catalysts are scarce and expensive, which is a serious drawback if the catalysts are to be used on a large scale. Earth-abundant elements-based catalysts should be considered for large-scale deployment. Iron is low-cost, environmentally friendly and high availability. Thus, iron-based water oxidizing catalysts are optimistic about being used in water–splitting systems^12–55^.

Although oxidation of water by FeCl_3_ was suggested thermally and photochemically many years ago^37^, the first report on water oxidation by iron compounds in the presence of an oxidant was reported by Elizarova *et al*. in 1981^36^. The research group observed water oxidation by FeCl_3_ and [Fe_2_O(Phen)_4_]Cl_4_, (Phen: 1,10-Phenanthroline), in the presence of [Ru(bpy)_3_]^3+^. During the reaction pH changes from 10 to 4.5. It is important to note that iron(II) and (III) ions are labile^56^ in metal complex and iron oxide is formed at pH ≥ 7. Thus under the condition of Elizarova’s reaction, iron oxide could be a true catalyst for water oxidation. Although, the stability of [Ru(bpy)_3_]^3+^ at pH 7 should also be checked because even low amounts of RuO_2_ from the decomposition of [Ru(bpy)_3_]^3+^ could efficiently catalyze water oxidation. Parmon *et al*. used Fe(OH)_3_ in the presence of [Ru(bpy)_3_]^3+^ at pH 10-11 and showed high activity toward water oxidation^38^. The used Fe(OH)_3_ was prepared by conventional procedures^39^, and it may not be optimized for water oxidation. Iron oxides with a higher activity for water oxidation could be prepared by other methods. Especially, now it is found that nanoparticles of iron oxides show efficient activity for water oxidation^57^. However, one investigation indicated that iron oxide is not formed and unlike to be a catalyst under acidic conditions^18^. Other investigations show that different phases of iron oxides are usually catalysts for water oxidation under non-acidic conditions^12–55^. Although under acidic conditions and in the presence of an iron complex, iron oxide is not a true catalyst^18^, FeO_4_^2−^ oxidizes water in acidic condition, but it could not perform water oxidation catalytically^40^.

In 2010, Bernhard and Collins reported a Fe-centered tetra amido macrocyclic ligand that efficiently catalyzes water oxidation (turnover frequency (TOF): 1.3 s^−1^), but a low turnover number ((TON) = 16) in the presence of a chemical oxidant (cerium ammonium nitrate (CAN)) was observed for the complex^58^. In 2011, Fe complexes with N-donor ligands were reported that efficiently catalyzed water oxidation with high efficiency for hours (turnover numbers more than 350 at pH = 1 and more than 1000 at pH = 2^14^. Using different methods, it was indicated that the Fe complex is a the true catalyst for water oxidation.

The catalytic activity of a few Fe complexes with N-donor ligands was investigated towards water oxidation at pH = 7–9. The study was performed by chemical methods, using [Ru(bpy)_3_](ClO_4_)_3_ (bpy = bipyridine) as an oxidant and showed the simple uncomplexed Fe(III) ion is even more active than the other Fe complexes under these conditions^16^. In the presence of [Ru(bpy)_3_](ClO_4_)_3_, the group proposed that Fe complexes are decomposed under oxidizing conditions to metal oxides, which form the true catalysts for water oxidation^16^. The Fe oxide particles formed in the water-oxidation conditions were isolated and detected to be Fe_2_O_3_ by the use of various techniques^16^. The results Fe complexes in the presence of Ce(IV) and [Ru(bpy)_3_](ClO_4_)_3_ suggest that the active Fe catalysts for water oxidation at acidic and basic conditions are different^16^. At acidic conditions, water oxidation by the Fe complexes appears to go through a molecular-based intermediate, with no Fe oxide formation. On the other hand, at basic conditions, Fe_2_O_3_ is the true catalyst for water oxidation^16^.

For water oxidation by some iron complexes in the presence of cerium(IV), Codola’ *et al*. reported a Fe^IV^–O–Ce^IV^ adduct, which showed cerium(IV) is not a simple and an innocent one-electron oxidant during the water oxidation reaction^12^.

Kottrup and Hetterscheid studied an on-line mass spectrometry approach to determine the onset of water oxidation and to determine between competing reactions oxygen evolution and carbon dioxide formation^28^. However, it is important to note that decomposition necessarily does not form carbon dioxide. Carbon dioxide is the product of deep oxidation of the ligand. Thus, decoordination of the ligand from complex could not be detected on-line mass spectrometry^28^.

Under acidic condition, the nature of α-[Fe(OTf)_2_(mcp)] (mcp = N,N′-dimethyl-N,N′-bis(pyridin-2- ylmethyl)cyclohexane-1,2-diamine, OTf = trifluoromethanesulfonate anion), under water oxidation and in the presence of cerium(IV) was investigated. Mössbauer spectroscopy showed that the complex produces oxo iron(IV)^31^. Using HPLC, the decomposition of the catalysts have been studied by identifying ligand fragments that form upon decomposition^31^. This analysis corresponds to the water oxidation activity of this catalyst with stability against degradation of the ligand *via* aliphatic C-H oxidation. This finding has served for the synthesis of a catalyst where sensitive C-H bonds have been replaced by C-D bonds^31^. Deuterated analog, displayed substantially more robust towards oxidative degradation and yields more than 3400 turnover frequency (TON). The information provides evidence that the water oxidation by a molecular structure than iron oxides^31^. Thus for many iron-based complexes, at low pH values, the true catalyst has molecular-based and in higher pH true catalyst has iron (hydr)oxide-based structures. It seems that to synthesize a stable iron complex under water-oxidation condition at higher pH (≥6), the stability of both ligand and complex is important. The stability of the ligand is not enough because even a stable ligand does not grantee the stability of the complex because the decoordination of the ligand from metal ion without degradation of the ligand is possible^31^.

In 2016, Masaoka’s group reported a pentanuclear iron complex with 3,5-bis(2-pyridyl)pyrazole ligand, which catalysis water oxidation with a turnover frequency of 1,900 per second^52^. In the structure, five iron ions are ligated by six dinucleating ligands, where three metal ions are also strongly electronically coupled via an oxo-bridge^52^. In acetonitrile, the complex exhibits four reversible one-electron oxidation-reduction steps corresponding to Fe(II)/Fe(III) couples, as shown by cyclic voltammetry. When water was added to the acetonitrile, a large electrocatalytic wave was seen at the potential corresponding to the fourth one-electron oxidation process, which is related to the catalytic water oxidation^52^. The research group proposed that the pentacoordinate equatorial iron centers of the complex provide two adjacent sites available for water binding and activation, which leads to pre-organized O-O bond formation^52^.

**Figure 1:**
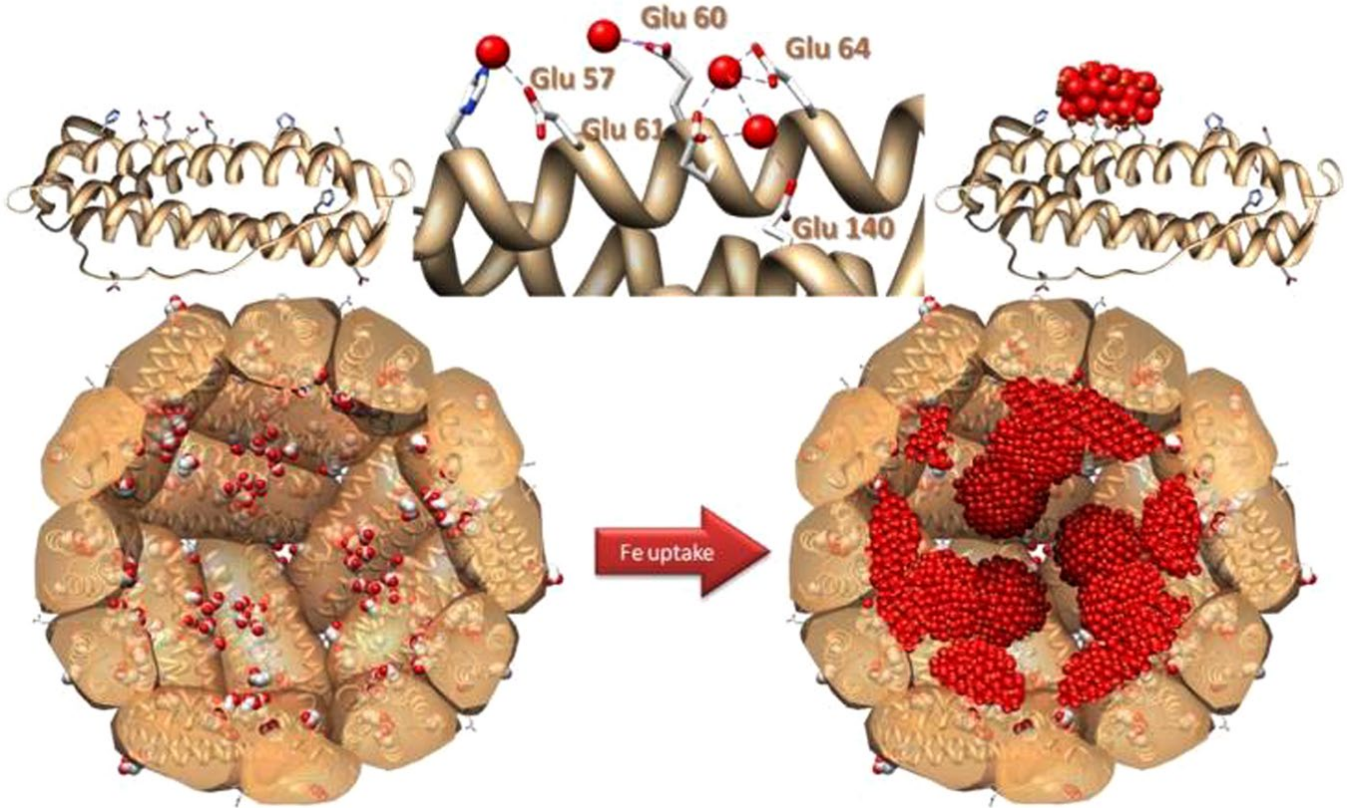
Nucleation sites (top) and schematic representation of the formation of the first iron clusters. Ferritin core growth (bottom): from the nucleation sites to the final iron oxide nanoparticle. The image and caption were from ref.^59^. Copyright (2013) by Elsevier.

As it was noted, iron oxides are an important iron-based catalyst for water oxidation. Ferritin (Fig. 1), the most important molecule participated in iron storage, storages non-heme iron. Ferritin is a globular protein (12 nm in diameter) with a central cavity (6 nm diameter)^59–62^. Iron is stored as hydrated iron oxide in the central cavity. The protein shell has a complicated structure with 24 subunits^59–62^.

## Results

Transmission electron microscopy (TEM)) images of ferritin showed nanosized iron oxides (ca. 4–6 nm) in the protein structure.

An iron-based core is not significantly larger than 8 nm independent of the different morphologies, as previously reported implies that the ferritin cavities have an inner diameter no greater than 8 nm^59–62^.

**Figure 2:**
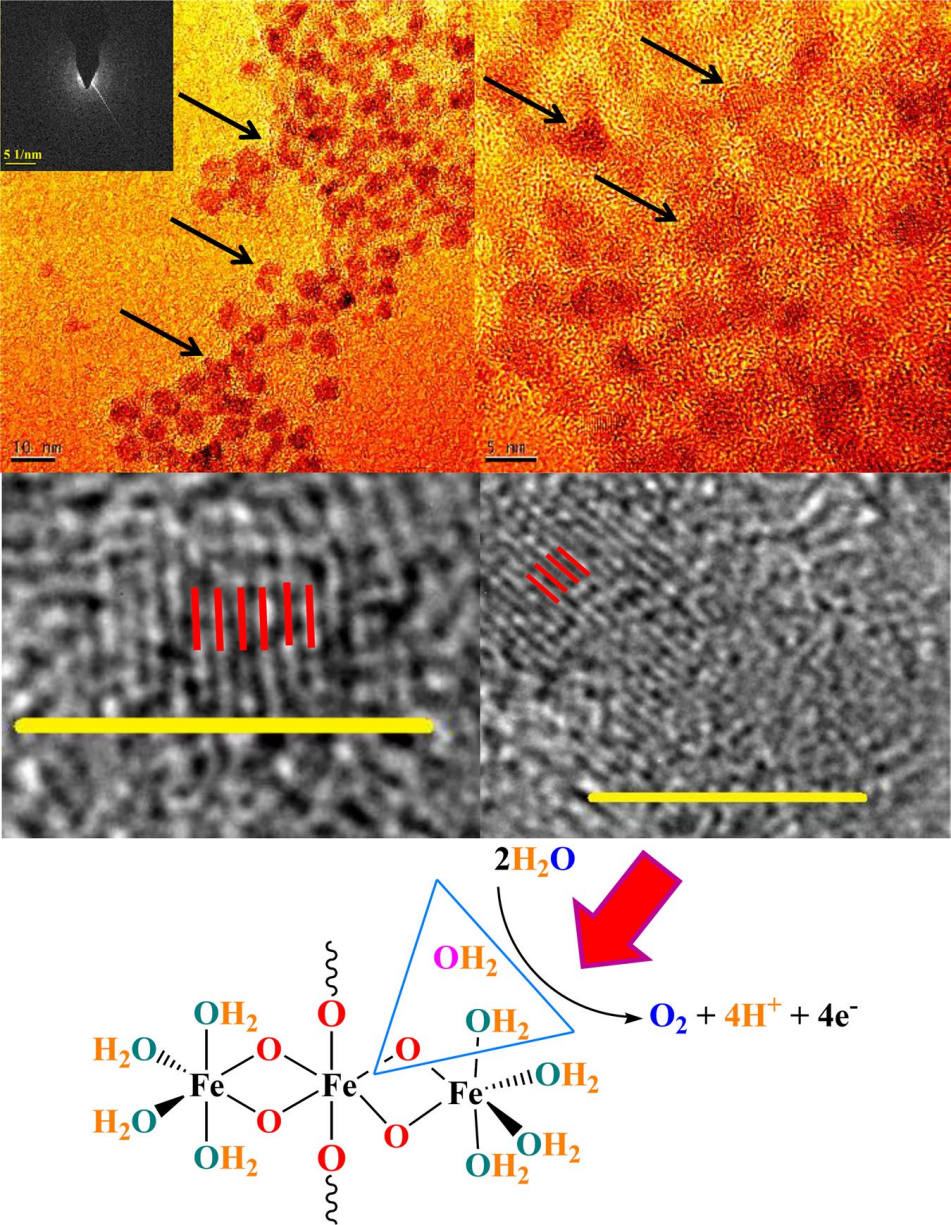
(HR)TEM images (**a**–**d**) of ferritin from the equine spleen (Scale bar for the image a is 10 nm; scale bar for the image **b**–**d** are 5 nm). SAED (e: scale bare: 1/5 nm) showed that the compound has a low crystalline. Red particles show the iron oxide section of ferritin. The black arrows show nanosized iron oxide. The red lines indicate d-spacings of 2.5–2.6 Å, which is corresponded to d-spacings of ferrihydrite crystal structure. Image a and b are colored forms obtained with the ImageJ software. We thank the ImageJ group (W. S. Rasband, ImageJ, U. S. National Institutes of Health, Bethesda, Maryland, USA, http://imagej.nih.gov/ij/, 1997–2016) for the use of the software. Schematic image shows a proposed mechanism for water oxidation by ferrihydrite in the structure of Ferritin.

High-resolution transmission electron microscopy (HRTEM) image of the iron core crystal structure indicates d-spacings of 2.5–2.6 Å, which is corresponded to d-spacings of ferrihydrite crystal structure (Fig. 2)^59–63^. Selected area (electron) diffraction (SAED), as crystallographic experimental methods, indicates diffuse rings attributed to a low crystalline compound.

A proposed mechanism for water oxidation in the presence of ferrihydrite of ferritin is shown in Fig. 2.

**Figure 3:**
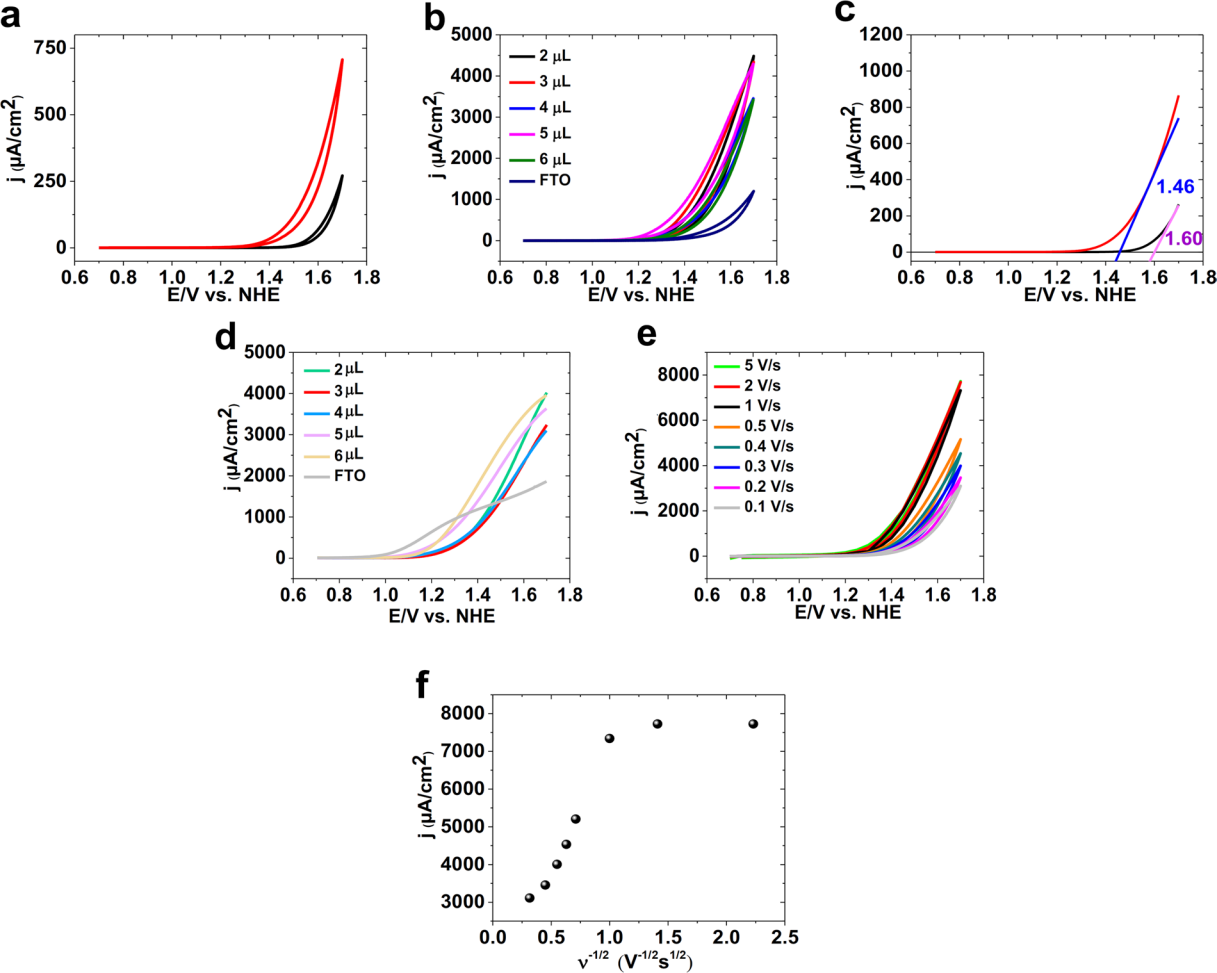
CVs for ferritin (red; 3.0 μM) and a bare FTO (black) (**a**) in the mixture of phosphate buffer (0.25 M, pH = 11.0) and scan rate 10.0 mV/s. CVs for different amounts of ferritin (**b**) in the mixture of phosphate buffer (0.25 M, pH = 11.0) and scan rate 100.0 mV/s. LSV for ferritin (red; 3 μM) and a fresh FTO (**c**) in the mixture of phosphate buffer (0.25 M, pH = 11.0) and scan rate 10.0 mV/s. SWV (amplitude: 100 mV; frequency: 10 Hz) for different amounts of ferritin (**d**) in the mixture of phosphate buffer (0.25 M, pH = 11.0). CV for ferritin at different scan rates in the mixture of phosphate buffer (0.25 M, pH = 11.0) (**e**). The plot of j vs. scan rate (**f**). Ferritin was placed on the FTO by Nafion. 3.0 μL or the reported amount of ferritin was dripped on the surface of an FTO and dried at room temperature, then 1.6 μL of 0.5 wt % Nafion solution was placed onto the surface of the electrode.

The cyclic voltammetry (CV) for ferritin was performed at pH = 11 (Fig. 3a,b; all potentials are reported vs. the normal hydrogen electrode (NHE)). No especial peak was observed for ferritin even at different concentrations (Fig. 3a,b).

As shown in Fig. 3c, ferritin is active for water oxidation compared to a bare FTO. Linear sweep voltammetry (LSV) shows the onset of the water-oxidation reaction as 1.46 V and 1.60 V for ferritin and a bare FTO, respectively (Fig. 3c). Thus, LSV shows that the onset for water oxidation is 140 mV less for FTO in the presence of ferritin than the bare FTO. No peak was also indicated for ferritin in CV and LSV. It is not surprising because iron oxide shows no peak under this condition. LSV shows that at 1.70 V and using FTO electrode, the water oxidation is four times more in the presence of ferritin.

Square wave voltammetry, a useful method to detect details, showed no peak for ferritin (Fig. 3d). The effect of scan rates is shown in Fig. 3e,f. At higher scan rates (>1 V/s), the current density is constant (Fig. 3e,f).

**Figure 4:**
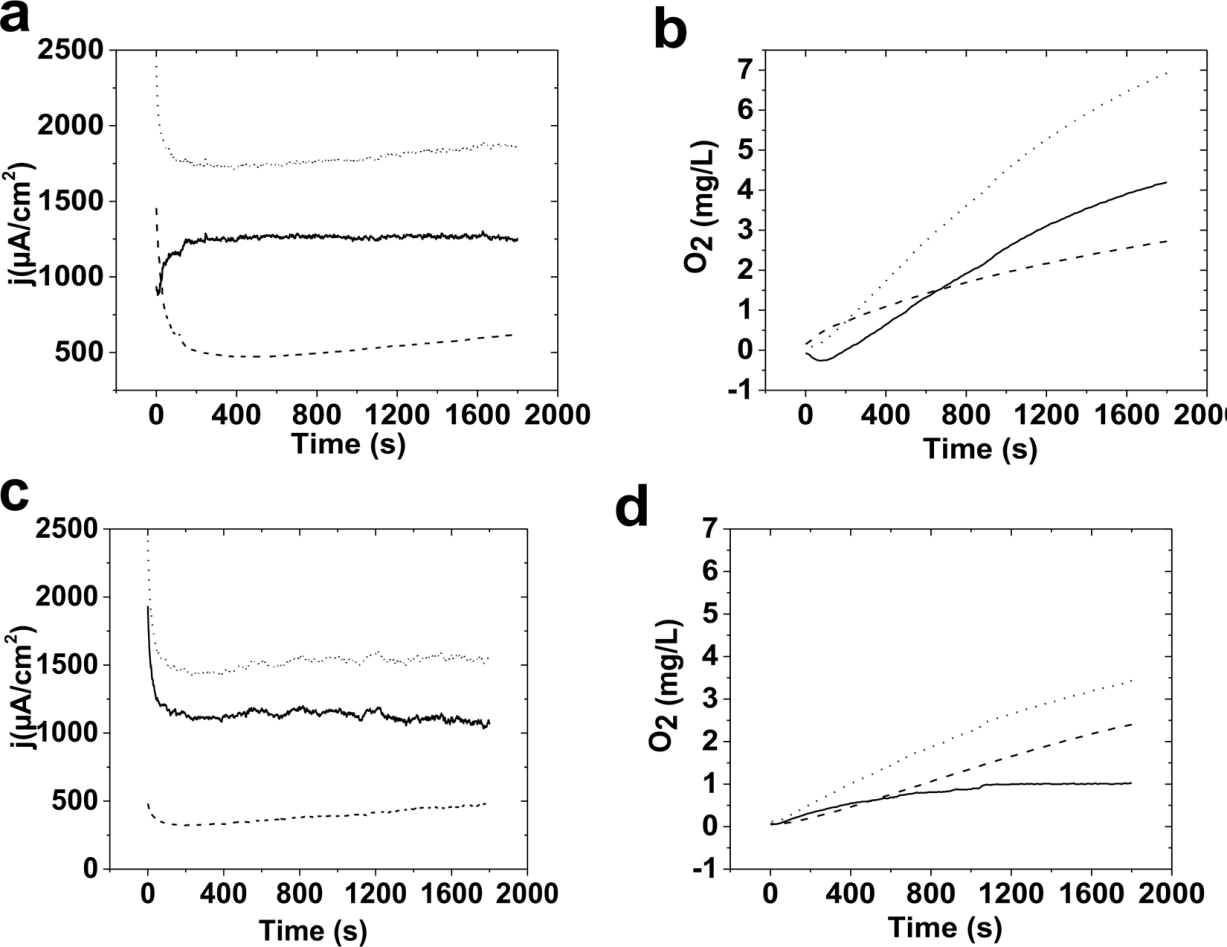
The amperometry (**a**) and oxygen evolution (**b**) for fresh ferritin (dot line) and a bare FTO (dash line) and subtraction of FTO on ferritin (solid line) at 1.7 V. The amperometry (**c**) and oxygen evolution (**d**) for ferritin effected by bacteria (dot line) and a bare FTO (dash line) subtraction of FTO from ferritin (solid line) at 1.7 V. Ferritin was placed on the FTO by Nafion. 3.0 μL of ferritin was dripped onto the FTO electrode surface and dried at room temperature, then 1.6 μL of 0.5 wt % Nafion solution was placed onto the surface of the electrode in the mixture of phosphate buffer (0.25 M, pH = 11.0) .

In the next step, we tested oxygen evolution by ferritin. As shown in Fig. 4a,b, oxygen evolution was observed for ferritin.

Assuming all iron ions are active for water oxidation, a turnover frequency of 0.001 s^−1^ at 1.7 V was calculated. FTO also indicated oxygen evolution under these conditions.

Streptococcus is proposed to be able to directly extract iron from ferritin, transferrin, lactoferrin, and hemoproteins, or indirectly by small siderophore molecules^64^. Siderophores (from the Greek: “iron carriers”) are relatively small molecules, which specifically chelates Fe(III) ions. These molecules are synthesized by bacteria and fungi under low iron stress^65^. Interestingly, the contaminated ferritin by bacteria (Staphylococcus^64^ and Streptococcus^66^ bacteria) shows lower activity than fresh ferritin, in fact, iron was consumed by the bacteria^64,66^, and evolution of oxygen was decreased due to the consumption (Fig. 4c,d).

It seems that in the absence of iron oxide, the decomposition of protein occurs under the harsh condition of water oxidation (Fig. 4c).

## Discussion

Recently, iron-based films have been investigated as electrocatalysts for water oxidation, especially Fe_2_O_3_ have been reported to be photocatalysts for water oxidation. Wu *et al*. reported iron oxides as a highly efficient catalyst for water oxidation^55^. The research group synthesized the catalyst on the surface of the electrodes from Fe(II) ions using simple cyclic voltammetry. As iron oxides are usually insulation, only a thin layer of iron oxide is an efficient catalyst for water oxidation^55^. An important finding is that the extremely low iron oxide is loading on the surface of the electrodes. Similarly, low amounts of iron oxide could be provided by ferritin for water oxidation^55^.

Water oxidation by metal oxides usually was reported at pH 13 or 14. However, a comparison for the modified electrode by ferritin at pH 11 by other metal oxides was indicated in Table S1.

The goal of this study is trying to overwhelm the limitations of natural and synthetic materials using a hybrid such as FTO/ferritin. Thus, it is a first step toward finding if a biomolecule such as ferritin could interface with FTO as a synthetic material toward the synthesize a semi-artificial electrode or not?^2^ Under our electrochemical conditions, the distance between iron oxides, which is placed in the enzyme’s pocket and the surface of the electrode causes a difficult way to the electron transfer. According to the location of the iron oxide in the chiral pocket of ferritin, such system is interesting to be used and developed efficient catalysts to oxidize and reduce both organic and inorganic substrates with a high selectively. For ferritin, at least a small size of iron oxide (6–8 nm) in a hydrophilicity/hydrophobicity pocket is interesting for a first-step investigation.

In addition to different strategies, which use iron-based or non-iron-based catalysts for water oxidation^12–55,67–75^, using a bimolecular structure is also interesting and could be a map road to future engineering artificial enzymes for water oxidation or other necessary reactions. Requirements for such hybrids are an increase in the compatibility of the electrode/enzyme, high porosity of the electrode, improvement in the electrode/enzyme interaction.

In summary, water-oxidation activity of ferritin from equine spleen was investigated. The biomolecule can be considered as nanosized iron oxides in a polymer matrix. The experiments showed that at pH 11, the compound is active for water oxidation with a turnover frequency of 0.001 s^−1^ at 1.7 V.

## Supplementary information

Supplementary information
