# Supplementary material for: Water oxidation by Ferritin: A semi-natural electrode
Source: Sci Rep. 2019 Aug 8;9:11499. doi: 10.1038/s41598-019-47661-z (PMC6687787; doi:10.1038/s41598-019-47661-z)
Supplement: Supplementary file 1 — Supplementary information [file 41598_2019_47661_MOESM1_ESM.doc]

**Supplementary Information**

**Water oxidation by Ferritin: A semi-natural electrode**

Zahra Abdi,^a^ Robabeh Bagheri,^b^ Zhenlun Song^b^ and Mohammad Mahdi Najafpour^*a, c, d^

*^a^Department of Chemistry, Institute for Advanced Studies in Basic Sciences (IASBS), Zanjan, 45137-66731, Iran*

*^b^* *Surface Protection Research Group, Surface Department, Ningbo Institute of Materials Technology and Engineering, Chinese Academy of Sciences, 519 Zhuangshi Road, Ningbo 315201, China*

*^c^Center of Climate Change and Global Warming, Institute for Advanced Studies in Basic Sciences (IASBS), Zanjan, 45137-66731, Iran*

*^d^Research Center for Basic Sciences & Modern Technologies (RBST), Institute for Advanced Studies in Basic Sciences (IASBS), Zanjan 45137-66731, Iran*

**Corresponding author; Phone: (+98) 24 3315 3201; E-mail:* [*mmnajafpour@iasbs.ac.ir*](mailto:mmnajafpour@iasbs.ac.ir)

**Experimental**

**Materials**

All reagents and solvents were purchased from Merck or Sigma-Aldrich Companies and were used without further purification. Fluorine doped tin oxide coated glass slide (FTO; surface resistivity ~7 Ω/sq), and Ferritin from equine spleen were purchased from the Sigma-Aldrich Company. The buffer solution was phosphate buffer (0.25 M, pH = 11.0).

**Methods**

High-resolution transmission electron microscopy (HRTEM and TEM) were investigated using an LEO 1430VP. Electrochemical experiments were carried out using an EmStat^3+^ device from the PalmSens Company (Netherlands). A three-electrode system was applied for the investigation of electrochemical properties. The Ag│AgCl and Pt electrode were used as reference and counter electrodes, respectively. Ferritin was placed on the FTO by Nafion; 3.0 μL of ferritin was dripped onto the FTO electrode surface and dried at room temperature, then 1.6 μL of 0.5 wt % Nafion solution was placed onto the surface of the electrode; then the modified FTO was used as working electrode.

In the paper, potentials were reported based on the NHE reference electrode. Oxygen evolution from aqueous solutions under amperometry at 25 ⁰C was investigated using an HQ40d portable dissolved oxygen-meter connected to an oxygen monitor with a digital readout.

**Characterization**

Electrochemical experiments were performed using an EmStat^3+^ from PalmSens (Netherlands). Voltammetry studies were carried out with a conventional three-electrode setup, in which FTO, Ag│AgCl, and a platinum foil served as working, reference, and auxiliary electrodes, respectively. All potentials in this project were reported vs. NHE. The distance between two opposite sides of the FTO electrode was measured by a digital caliper MarCal 16ER model (Mahr, Germany). The temperature was measured by Laserliner 082 (Germany).

**Oxygen evolution measurement**

The O_2_ measurement in solution was carried out at 25.0 ⁰C using an optical-probe oxygen meter (HQ40d from Hach, Düsseldorf, Germany) (Figure S1).


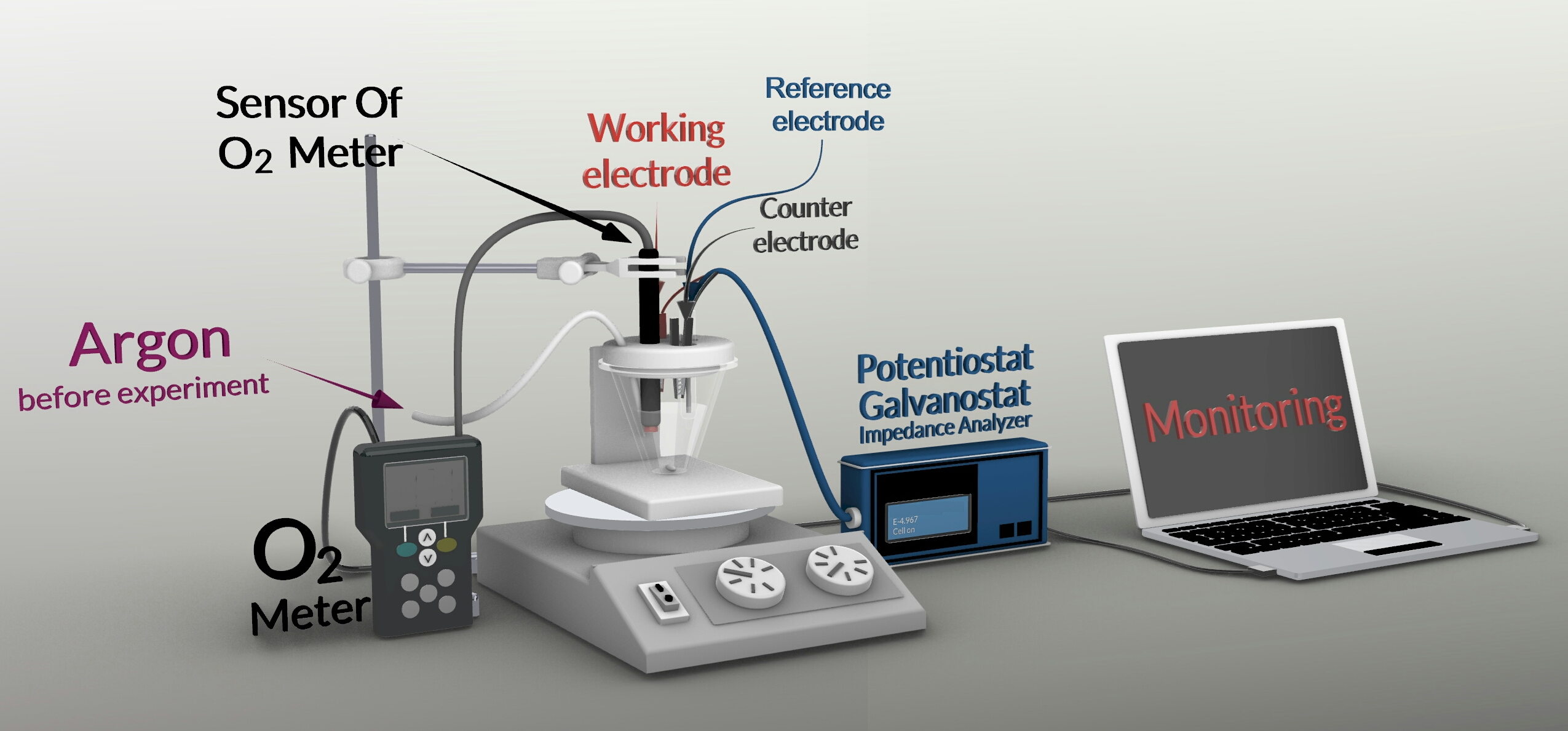


Figure S1 Set up for oxygen evolution/amperometry.


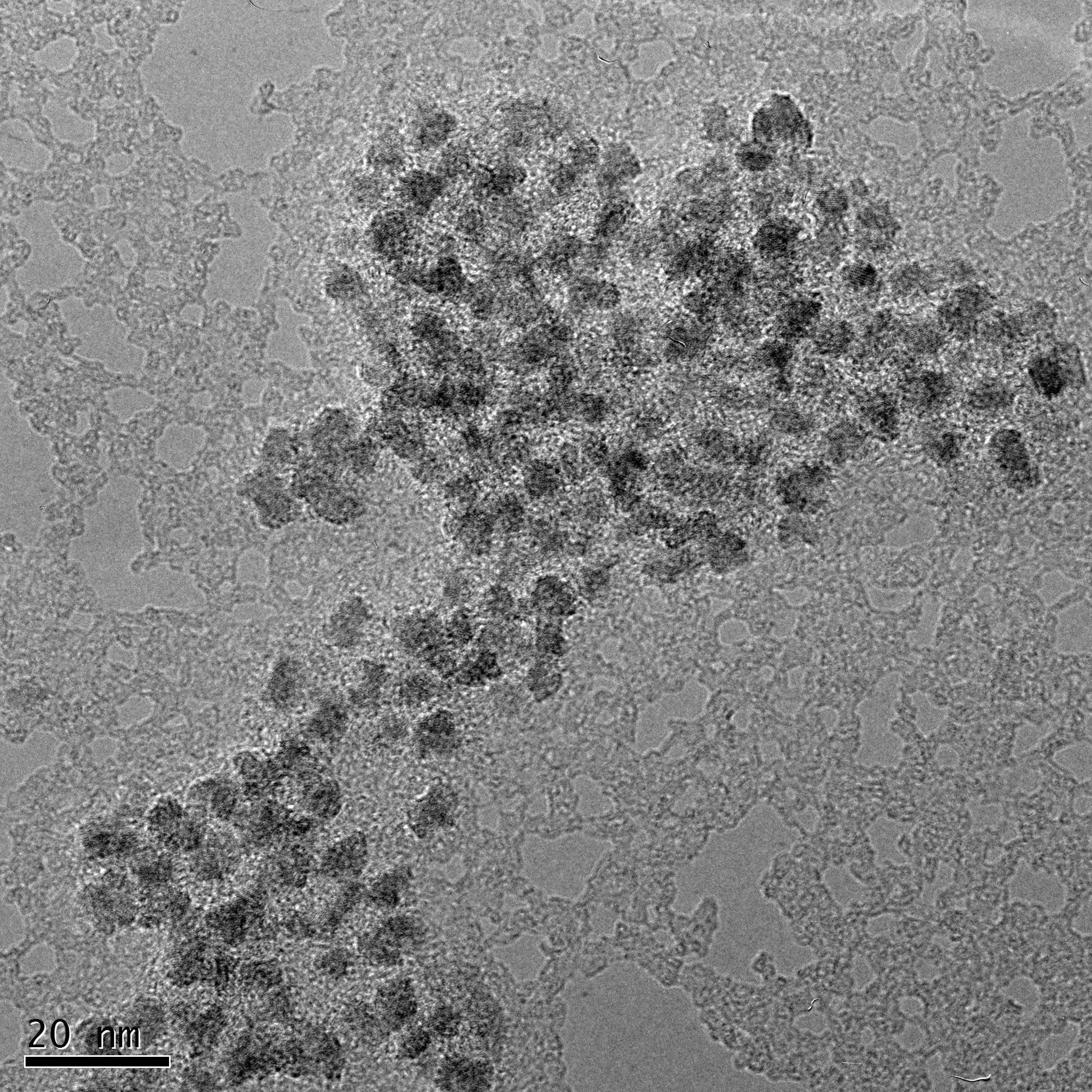

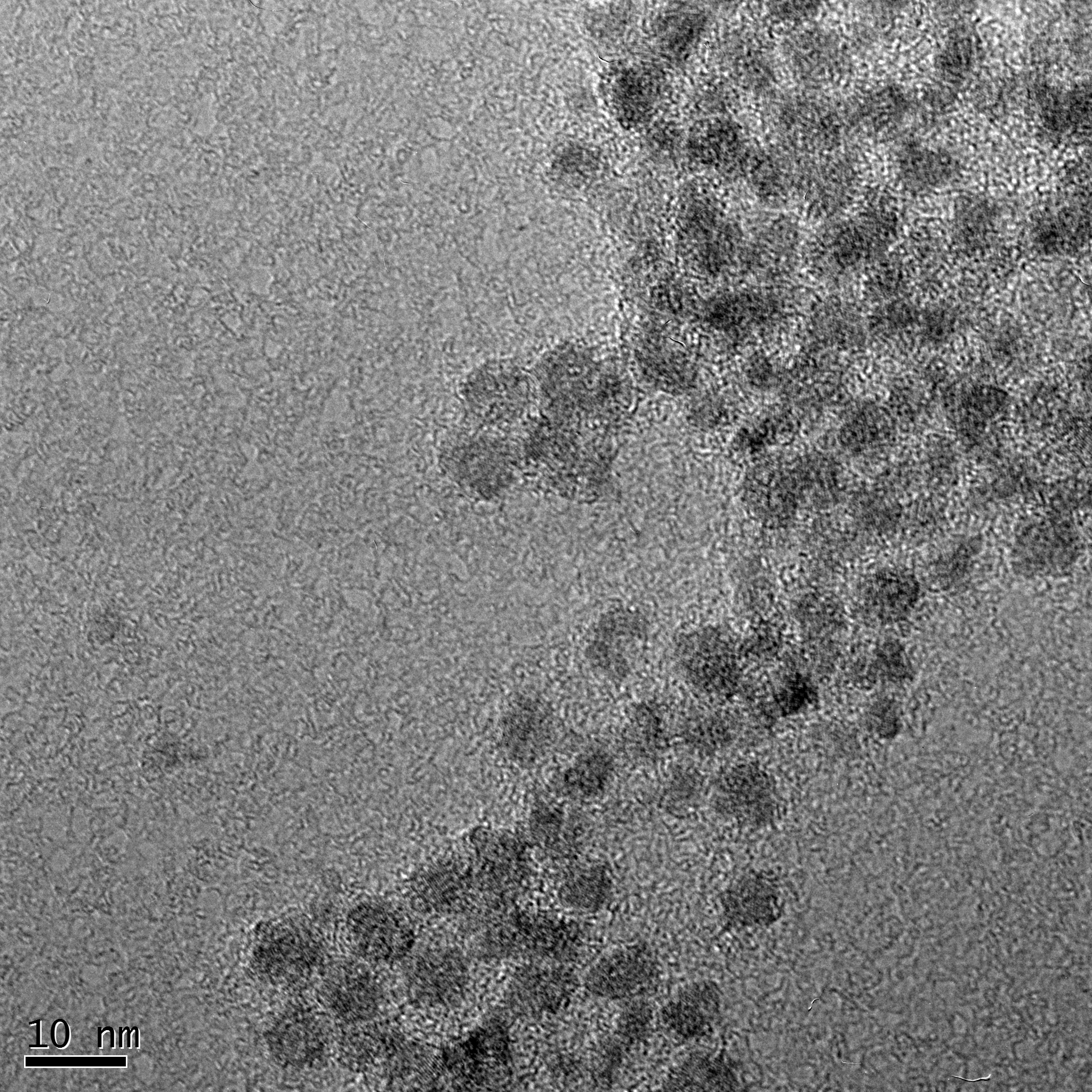

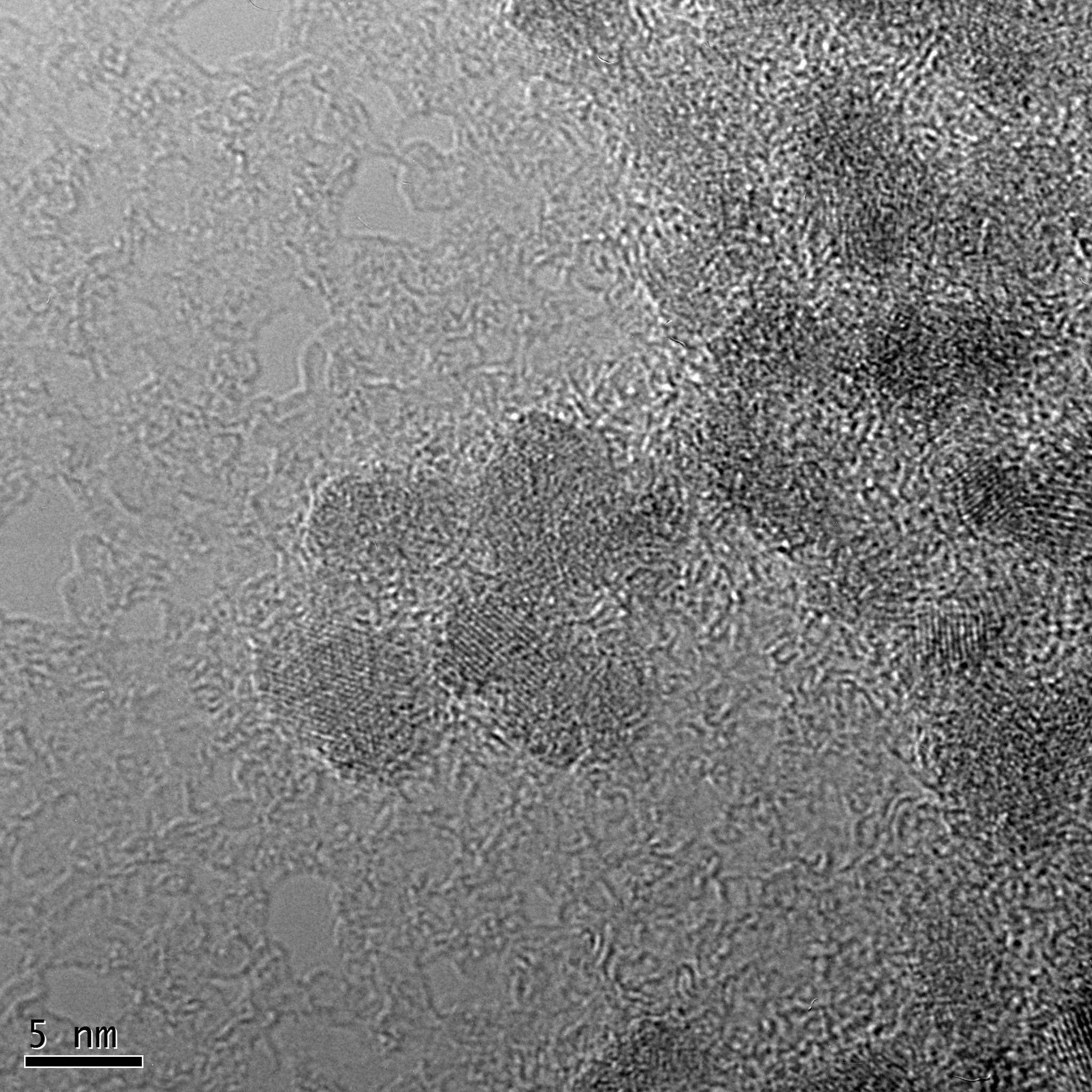

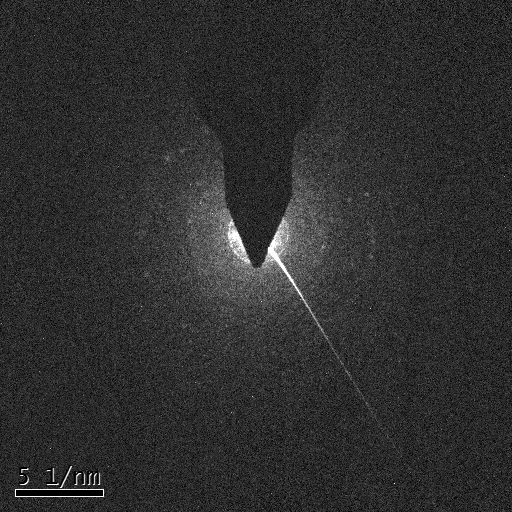


a

b

c

d

Figure S2 (HR)TEM images of ferritin from equine spleen with different magnifications (a-c). SAED showed that the compound is amorphous.

**Table S1** Comparison of some heterogeneous catalysts for water oxidation.

| **Comp.** | **η^[a]^ (mV)** | **η^[b]^ (mV)** | **pH** | **Ref.** |
| --- | --- | --- | --- | --- |

| **This work** | **11** | **̴ 1300** | **826** | **The modified electrode by** **ferritin** |
| --- | --- | --- | --- | --- |
| 1 | 14 | 297 | - | NiFeO_x_ |
| 2 | 14 | 445 | 345 | FeO_x_ |
| 1 | 14 | 405 | - | FeO_x_ |
| 3 | 14 | 430 | < 350 | Fe_2_O_3_ |
| 4 | 13 | - | 270 | Fe_3_Ni_2_O_x_ |
| 5 | 13 | - | 211 | FeNiO_x_ |
| 6 | 13 | 250 | 190 | Fe_2_Ni_3_O_x_ |
| 7 | 13 | - | 397 | CoFeO_x_ |
| 5 | 13 | 410 | 320 | FeO_x_ |
| 5 | 13 | - | 181 | FeCoO_x_ |
| 5 | 13 | - | 191 | FeCoNiO_x_ |
| 4 | 13 | - | 270 | Ni_2_FeAlO_x_ |
| 4 | 13 | - | 250 | NiFeMo_3_O_x_ |
| 4 | 13 | - | 240 | Ni_2_FeCr_2_O_x_ |
| 4 | 13 | - | 240 | NiFeGa_3_O_x_ |
| 8 | 11 | 420 | 300 | FeOOH |
| 9 | 7 | > 600 | 291 | CoFeO_x_ |

[a] Onset overpotential. [b] @1 mAcm^-2^.

1. Trotochaud, L.; Ranney, J. K.; Williams, K. N.; Boettcher, S. W., Solution-cast metal oxide thin film electrocatalysts for oxygen evolution. *J. Am. Chem. Soc.* **2012,** *134* (41), 17253-17261.

2. Doyle, R. L.; Lyons, M., Kinetics and mechanistic aspects of the oxygen evolution reaction at hydrous iron oxide films in base. *J. Electrochem. Soc.* **2013,** *160* (2), H142-H154.

3. Qiu, Y.; Leung, S. -F.; Zhang, Q.; Hua, B.; Lin, Q.; Wei, Z.; Tsui, K. -H.; Zhang, Y.; Yang, S.; Fan, Z., Efficient photoelectrochemical water splitting with ultrathin films of hematite on three-dimensional nanophotonic structures. *Nano Lett.* **2014,** *14* (4), 2123-2129.

4. Chen, J. Y.; Miller, J. T.; Gerken, J. B.; Stahl, S. S., Inverse spinel NiFeAlO_4_ as a highly active oxygen evolution electrocatalyst: promotion of activity by a redox-inert metal ion. *Energy Environ. Sci.* **2014,** *7* (4), 1382-1386.

5. Smith, R. D.; Prévot, M. S.; Fagan, R. D.; Zhang, Z.; Sedach, P. A.; Siu, M. K. J.; Trudel, S.; Berlinguette, C. P., Photochemical route for accessing amorphous metal oxide materials for water oxidation catalysis. *Science* **2013**, 1233638.

6. Smith, R. D.; Prévot, M. S.; Fagan, R. D.; Trudel, S.; Berlinguette, C. P., Water oxidation catalysis: electrocatalytic response to metal stoichiometry in amorphous metal oxide films containing iron, cobalt, and nickel. *J. Am. Chem. Soc.* **2013,** *135* (31), 11580-11586.

7.Abellán, G.; Carrasco, J. A.; Coronado, E.; Romero, J.; Varela, M., Alkoxide-intercalated CoFe-layered double hydroxides as precursors of colloidal nanosheet suspensions: structural, magnetic and electrochemical properties. *J. Mater. Chem. C* **2014,** *2* (19), 3723-3731.

8. Chemelewski, W. D.; Lee, H.-C.; Lin, J.-F.; Bard, A. J.; Mullins, C. B., Amorphous FeOOH oxygen evolution reaction catalyst for photoelectrochemical water splitting. *J. Am. Chem. Soc.* **2014,** *136* (7), 2843-2850.

9. Pintado, S.; Goberna-Ferrón, S.; Escudero-Adán, E. C.; Galán-Mascarós, J. R. n., Fast and persistent electrocatalytic water oxidation by Co–Fe Prussian blue coordination polymers. *J. Am. Chem. Soc.* **2013,** *135* (36), 13270-13273.
